# Dapsone in the Management of Pemphigus and Pemphigoid: Rediscovery of its Long-Lost Efficacy

**DOI:** 10.7759/cureus.8805

**Published:** 2020-06-24

**Authors:** Salim Alkeraye, Lama R AlZamil, Suha Alenazi

**Affiliations:** 1 Department of Dermatology, College of Medicine, King Saud University, Riyadh, SAU

**Keywords:** pemphigus vulgaris, dapsone, bullous pemphigoid, steroid sparing, blistering skin disease

## Abstract

Introduction

Autoimmune mucocutaneous blistering dermatoses (AMCBD) are a group of disorders characterized by the production of autoantibodies that target specific adhesion molecules of the skin and/or mucous membranes. As a result, there is blister formation on the skin with or without mucous membrane involvement. Systemic corticosteroids have been used as the mainstay of treatment in AMCBD. However, due to the debilitating side effects associated with their use, there is significant morbidity and mortality, especially on the fragile elderly patients. Although the efficacy of dapsone in the treatment of AMCBD was identified decades ago, few recent studies shed light on that. Hence, further studies are needed to evaluate the efficacy of dapsone as a single agent in maintaining disease remission in patients with AMCBD.

Materials and methods

An observational retrospective study was performed. Patients with a known diagnosis of bullous pemphigoid (BP) or pemphigus vulgaris (PV) who are treated with dapsone with or without low-dose systemic corticosteroids were included in the study, and their medical files were reviewed.

Results

A total of seven patients were included (three males and four females). All patients showed a satisfactory response to dapsone, achieving disease remission in a short period of time with no serious side effects necessitating treatment cessation.

Conclusions

Our findings support that dapsone may have a corticosteroid-sparing effect in the management of AMCBD. Further studies are warranted to confirm our findings.

## Introduction

Autoimmune mucocutaneous blistering dermatoses (AMCBD) are a group of disorders characterized by the production of autoantibodies that target specific adhesion molecules of the skin and/or mucous membranes. As a result, there is blister formation on the skin with or without mucous membrane involvement. The blisters range from superficial to deep and from loose, easily ruptured blisters to tense blisters depending on the variant of AMCBD. There are two main variants of AMCBD, which are pemphigoid and pemphigus groups and each group is divided into different subsets of diseases. Bullous pemphigoid (BP) is the most common autoimmune blistering skin disease. It arises as a consequence of autoantibodies attacking the hemidesmosome component of the basement membrane zone (BMZ) specifically BPAG1 (BP230) and BPAG2 (BP180), with the latter being predominately involved in the initial immune response due to its structure and location in the BMZ [1]. BP is characterized by an intensely pruritic eruption along with the formation of subepidermal tense blisters mainly on the trunk and flexural surfaces of the extremities [1,2]. It predominately affects the elderly population at the age of 60 to 80 years old. Moreover, there are various therapeutic options for BP and each treatment regimen must be tailored carefully according to the patient’s comorbidities and severity of the disease. Since decades systemic corticosteroids have been used as the mainstay of treatment in BP with recommended doses of 0.5-0.75 mg/kg/day [2]. However, due to the age group affected by BP, there is significant morbidity and mortality associated with the use of systemic corticosteroids. Rzany et al. concluded in their study that there is an increased case-fatality rate among patients with BP, especially those who required higher doses of systemic corticosteroids [3]. This emphasizes the harmful impact of systemic corticosteroids on the fragile elderly group with severe disease. In addition, adjuvant medications can be considered in the treatment of BP to obtain or maintain disease control such as immunosuppressants, which include azathioprine, methotrexate, ciclosporin, or mycophenolate mofetil, and antibiotics like dapsone and tetracycline+/- nicotinamide [2,4]. Dapsone is an antibacterial medication that belongs to the sulfonamide class of antibiotics. However, since the discovery of its anti-inflammatory properties, it has been used for the treatment of various skin conditions including AMCBD. Pemphigus vulgaris (PV) is the most common blistering disease among the pemphigus group and it accounts for 70% of all pemphigus cases [5,6]. Unlike BP, where mucous membranes are rarely involved [1], the disease process in PV involves both skin and mucous membranes, with the latter being mostly affected first [5]. The pathophysiology of PV is related to IgG autoantibodies against proteins of the desmosome complex specifically desmoglein (Dsg)1 and/or Dsg3, which hold keratinocytes together. As a result, the intraepithelial junctions are compromised and acantholysis subsequently occurs which is considered a characteristic sign for the pemphigus group of diseases [6]. PV most commonly affects patients between the ages of 40 and 60 years old. Clinically, it presents initially with painful oral erosions and other sites can be affected as well, such as the esophagus in which patients would complain of dysphagia. Skin involvement occurs later, and it is characterized by flaccid fragile blisters that easily rupture, leaving behind painful erosive areas that ooze and bleed easily later forming crusts. The cutaneous involvement can be either localized or generalized and is mostly seen on the trunk, groin, scalp, face, and extremities usually sparing the palms and soles [5,6]. Systemic corticosteroids are the basis of PV treatment with an initial dose of 1-2 mg/kg/day [7]. A recent study has recommended the use of rituximab as a first-line agent in addition to short-term prednisone in the treatment of PV, which showed higher efficacy and fewer side effects than systemic corticosteroids alone [8]. Additionally, intravenous immunoglobulin (IVIG) has been shown to be effective in the treatment of AMCBD including both BP and PV usually as a third-line adjunct in refractory cases [7,9]. Moreover, several types of adjuvant medications are available for PV, including dapsone with the aim of reducing side effects of systemic corticosteroids or in case of the presence of a contraindication preventing their use [7]. Although the efficacy of dapsone in the treatment of AMCBD was identified decades ago, few recent studies shed light on that. Hence, further studies are needed to evaluate the efficacy of dapsone as a single medication in maintaining disease remission in patients with AMCBD.

## Materials and methods

The study is an observational retrospective study of patients attending the dermatology clinic at King Khalid University Hospital (KKUH), which is a tertiary care hospital in Riyadh, Saudi Arabia. The study’s proposal was ethically approved by the Institutional Review Board (IRB) of College of Medicine at King Saud University, IRB no. E-20-4630. Patients selection was based on the following inclusion criteria: age ≥18 years old, confirmed cases of pemphigoid or pemphigus who are treated with dapsone alone or as an adjunct to low dose systemic corticosteroids (i.e., ≤7 mg/day). The exclusion criterion was patients who are on a high dose of systemic corticosteroids (i.e., ≥ 7 mg/day). The primary outcome measure was the response to dapsone in either inducing disease remission or maintaining it. The data were extracted from the electronic medical records at KKUH using a data collection form. The form consisted of two main parts, the first part was for describing demographics and the patient’s disease and treatment characteristics such as previous medications and their duration and any concomitant immunosuppressive treatment. The second part was for assessing the response to dapsone, the dose of dapsone, and any side-effects of the medication.

## Results

**Figure 1:**
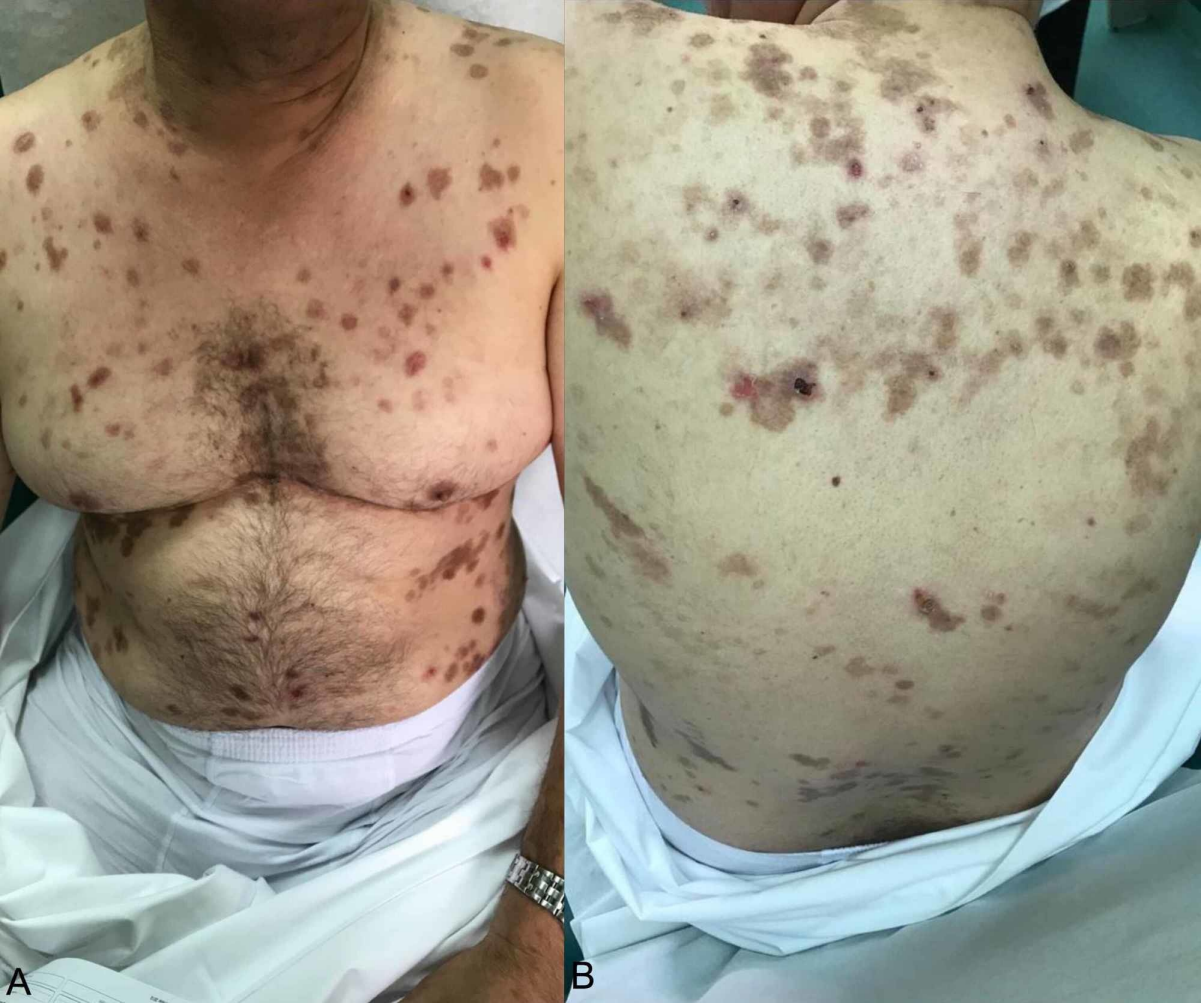
(Patient No. 7) Crusted erosions and blisters on the chest, abdomen, and upper back

**Figure 2:**
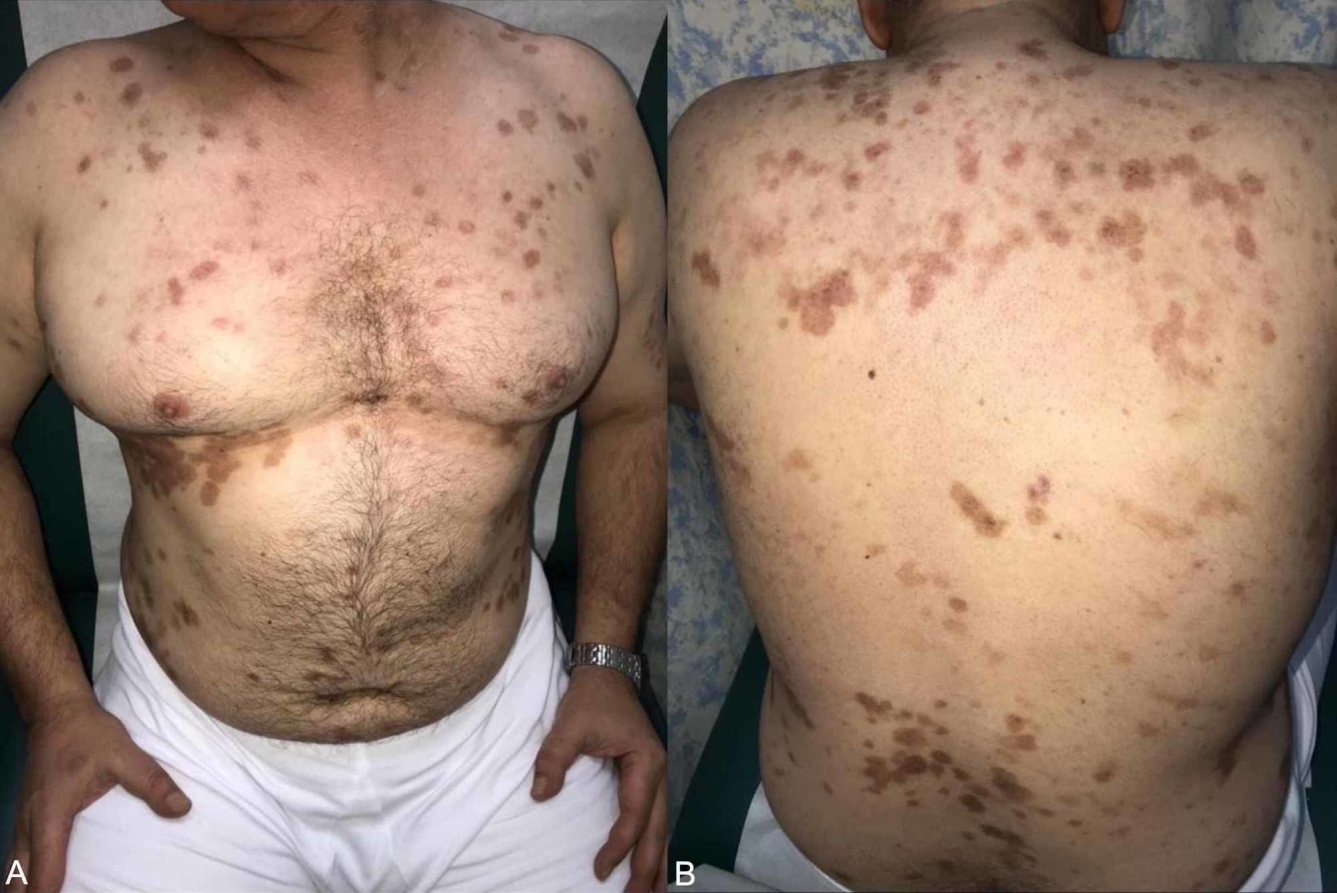
(Patient No. 7) Post-inflammatory hyperpigmented macules and patches on the chest, abdomen, and back

A total of seven patients who met the inclusion criteria were included. Three male and four female patients whose ages range from 24 to 80 years old have been treated with dapsone for pemphigus or pemphigoid disease (Table 1). All but one patient have received oral and/or topical corticosteroids prior to the use of dapsone. Although the severity of the disease varies among those patients, they all showed a 100% response to doses of 50 to 100 mg of dapsone. Patient number (No.) 1 is a 33-year-old female who had been treated with dapsone for 18 months and achieved complete resolution of her disease. Patient No. 2 is a 32-year-old female who had been treated with dapsone for two years and maintained clinical remission for 18 months off treatment. Patient No. 3 is an 80-year-old female who had been treated with dapsone for two years and maintained remission for 18 months off treatment. Patient No. 4 is a 60-year-old female who had been treated with dapsone for 16 months and maintained remission for 12 months off treatment. Patient No. 5 is a 53-year-old male who had been treated with dapsone for 18 months and maintained remission for 12 months off treatment. Patient No. 6 is a 24-year-old male who had been treated with dapsone for three years and continued to show clinical remission for two years after treatment cessation. Patient No. 7 is a 45-year-old male who was diagnosed with pemphigus foliaceus that involved 20% of the total body surface area with no mucosal involvement (Figure 1A, 1B). Histopathologically, sections from biopsy for direct immunofluorescence (DIF) of perilesional skin showed positive intercellular IgG and C3 mainly in the superficial layers of the epidermis. The patient had been treated with 100 mg of dapsone for six months and achieved clinical remission off treatment (Figure 2A, 2B). No serious side-effects were noted in any of the patients in our cohort.

**Table 1:** Clinical data of pemphigus and pemphigoid patients

Patient No.	Gender	Age	Diagnosis	Previous medications
1	Female	33	Pemphigus vulgaris	Oral corticotherapy (0.5mg/kg/day) for two months
2	Female	32	Pemphigus vulgaris	Topical corticotherapy for five months
3	Female	80	Bullous pemphigoid	Oral corticotherapy (0.5 mg/kg/day) for two months
4	Female	60	Bullous pemphigoid	None
5	Male	53	Bullous pemphigoid	Oral corticotherapy (0.5 mg/kg/day) for one month
6	Male	24	Pemphigus vulgaris	Oral corticotherapy (0.5 mg/kg/day) for six weeks
7	Male	45	Pemphigus foliaceus	Oral corticotherapy (0.5 mg/kg/day) for two years

## Discussion

Dapsone or 4,4'-diaminodiphenylsulfone is a chemotherapeutic agent that belongs to the sulfonamide class of antibiotics. It was first used in 1945 for the treatment of leprosy (or Hansen's disease) [10], and it is currently listed on the World Health Organization’s (WHO) model list of essential medicines as part of the multidrug therapy for the treatment of leprosy [11]. In addition, it is used in the treatment of other infections like malaria, toxoplasmosis, and pneumocystis carinii pneumonia [12,13]. In 1950, dapsone was identified to exhibit an anti-inflammatory effect in dermatitis herpetiformis, thereafter its use in skin disorders began [10]. It has been discovered that the anti-inflammatory action of dapsone lies in its ability to suppress neutrophils migration and production of toxic secretory products which damage the skin [10,14]. Therefore, it has been shown to be effective in various skin disorders associated with abnormal neutrophils accumulation such as dermatitis herpetiformis, linear IgA bullous dermatosis, pyoderma gangrenosum, PV, BP, sweet’s syndrome and vasculitis [10,13]. Nowadays dapsone is mainly used as an adjuvant to systemic corticosteroids, and there are multiple studies which described its effectiveness as an adjuvant treatment to corticosteroids such as the study by Sticherling et al. who recommended adjuvant dapsone as the preferred steroid-sparing agent over azathioprine in the treatment of moderate to severe BP due to the higher efficacy and safety of dapsone over azathioprine [15]. Conversely, another study showed no significant difference between azathioprine and dapsone as adjuvants in the treatment of BP and reported that they are both equally effective [12]. Over the past years, several authors have pointed out the beneficial effects of dapsone as a single agent in the treatment of different skin diseases in a considerable number of reports and clinical studies. It was first discovered by Esteves and Brandao et al. in 1950 who reported the effectiveness of dapsone in the treatment of dermatitis herpetiformis, which is a chronic autoimmune blistering disease characterized by an exquisitely pruritic vesicular eruption and it is closely related to celiac disease [16]. Since that time dapsone became the drug of choice for patients with dermatitis herpetiformis (besides gluten-free diet which needs time to exhibit its effect on skin [17]) especially during the first few months following diagnosis given its effectiveness in reducing itchiness within 24 hours and regression of cutaneous lesions within one week approximately [17-21]. Similarly, in 1976, Piamphongsant et al. reported a series of three uncomplicated cases of BP which were treated with dapsone alone, the patients were aged 25, 30, and 70 years old. All three cases showed good response to dapsone, achieving clinical improvement within one to two weeks of initiation of treatment [22]. Moreover, a retrospective study of 28 patients with BP was done in 1983, which showed complete response to dapsone when used as a single agent in the treatment of 12 cases, this was achieved over two to three weeks of initiation of treatment. Additionally, in the same study, one case was initially treated with prednisolone and dapsone, however, after four months, prednisolone was discontinued and dapsone alone was continued which successfully maintained control of the disease [23]. On the contrary, in the same study, the authors had also reported “no response” to dapsone in three cases; one of them was a two-year-old child, this group of “non-responders” was given similar doses of dapsone as in the responders' group; however, the drug was only given for one week and then discontinued as there was no clinical improvement. That raises the question about the possibility of the treatment being effective in this group only if it was continued for a longer period. Another study was conducted on 36 BP patients; 15 patients received dapsone alone (group 1) and 19 patients were treated with dapsone plus topical steroids (group 2). The study showed no significant difference between the two groups, although, the authors suggested that a combination of dapsone and topical steroids is more effective than dapsone alone. Despite that, failure of treatment was observed in 20 cases even though one patient was receiving dapsone and systemic steroids [24]. Likewise, a study on 18 patients with BP reported a total of eight patients who responded completely to dapsone as the sole treatment, while 6 patients showed partial response and four patients did not respond at all. All patients in that study were also using topical steroids as needed. Four patients of the complete responders were gradually withdrawn from dapsone after achieving complete control of their disease and still remained in remission after a follow-up period of nine months to two years [25]. Additionally, the authors of the previous study did not find an absolute correlation between the dense neutrophilic infiltration on histopathological sections and the response to dapsone. In fact, two of their patients had substantial neutrophils infiltration but only partially responded to dapsone or did not respond at all. And on the other hand, one patient responded completely to dapsone although he/she had no neutrophils infiltration. Furthermore, dapsone was also noted as effective in multiple cases of PV [26-30]. A series of three cases were reported by Piamphongsant et al. each having distinct clinical scenarios that all responded to dapsone. Case No. 1 was diagnosed with pemphigus foliaceus and successfully treated by dapsone 100 mg/day and the dose was gradually tapered until reaching 20 mg/day with no active lesions. Additionally, case No. 2 was a patient with pemphigus erythematosus who was on prednisone and cyclophosphamide, however, the patient developed hepatoxicity necessitating discontinuation of cyclophosphamide. Prednisone 15 mg/day was unable to control the disease, therefore, dapsone 100 mg/day was added which successfully controlled the disease over a follow-up period of four months. Case No. 3 was a PV patient on 60 mg/day prednisone, the patient underwent surgery and was having problems with the healing of the incision wound, immediately prednisone was tapered to 10 mg/day and dapsone 100 mg/day was added. The wound healed and no active lesions were reported over a follow-up period of six months [26]. Dapsone has a variety of recognized adverse events; however, they are dose-dependent, reversible, and most are unnoticed by the patient. The most frequent side-effects are of a hematological nature, including methemoglobinemia, hemolytic anemia; especially in G6PD-deficient patients who are more susceptible to hemolysis and less susceptible to methemoglobinemia, and abnormal liver function tests [13]. Other rare side-effects of dapsone include agranulocytosis, peripheral neuropathy, and “dapsone syndrome” which are all assumed to be of an idiosyncratic or unknown etiology [10]. In general, these side-effects of dapsone are considered very low if the plasma concentration is kept below 5 mg/L [30]. Our data support the reported results. All our patients showed a satisfactory response to dapsone achieving disease remission in a short period of time with no serious side-effects necessitating treatment cessation.

## Conclusions

The study aimed to shed light on an effective alternative treatment to systemic corticosteroids, which in the long term, have debilitating side-effects, especially for elderly patients. Our findings support that dapsone may have a corticosteroid-sparing effect in the management of AMCBD. Further studies are warranted to confirm our findings.

## References

[1] Miyamoto D, Giuli Santi C, Aoki V, Wakisaka Maruta C (2019). Bullous pemphigoid. An Bras Dermatol.

[2] Feliciani C, Joly P, Jonkman MF (2015). Management of bullous pemphigoid: the european dermatology forum consensus in collaboration with the european academy of dermatology and venereology. Br J Dermatol.

[3] Rzany B, Partscht K, Jung M (2002). Risk factors for lethal outcome in patients with bullous pemphigoid: low serum albumin level, high dosage of glucocorticosteroids, and old age. Arch Dermatol.

[4] Yancey KB, Egan CA (2000). Pemphigoid: clinical, histologic, immunopathologic, and therapeutic considerations. JAMA.

[5] Joly P, Litrowski N (2011). Pemphigus group (vulgaris, vegetans, foliaceus, herpetiformis, brasiliensis). Clin Dermatol.

[6] Porro A, Seque C, Ferreira M, Enokihara M (2019). Pemphigus vulgaris. An Bras Dermatol.

[7] Harman KE, Brown D, Exton LS (2017). British association of dermatologists’ guidelines for the management of pemphigus vulgaris 2017. Br J Dermatol.

[8] Joly P, Maho-Vaillant M, Prost-Squarcioni C (2017). First-line rituximab combined with short-term prednisone versus prednisone alone for the treatment of pemphigus (Ritux 3): a prospective, multicentre, parallel-group, open-label randomised trial. Lancet.

[9] Hoffmann JHO, Enk AH (2019). High-dose intravenous immunoglobulin in skin autoimmune disease. Front Immunol.

[10] Zhu YI, Stiller MJ (2001). Dapsone and sulfones in dermatology: overview and update. J Am Acad Dermatol.

[11] (2020). World Health Organization Model List of Essential Medicines, 21st List, 2019. Geneva: World Health Organization; 2019. Licence: CC BY-NC-SA 3.0 IGO.. https://www.who.int/medicines/publications/essentialmedicines/en/.

[12] Tirado-Sánchez A, Díaz-Molina V, Ponce-Olivera RM (2012). Efficacy and safety of azathioprine and dapsone as an adjuvant in the treatment of bullous pemphigoid. Allergol Immunopathol.

[13] Wozel G, Blasum C (2014). Dapsone in dermatology and beyond. Arch Dermatol Res.

[14] Debol SM, Herron MJ, Nelson RD (1997). Anti-inflammatory action of dapsone: Inhibition of neutrophil adherence is associated with inhibition of chemoattractant-induced signal transduction. J Leukoc Biol.

[15] Sticherling M, Franke A, Aberer E (2017). An open, multicentre, randomized clinical study in patients with bullous pemphigoid comparing methylprednisolone and azathioprine with methylprednisolone and dapsone. Br J Dermatol.

[16] Esteves J, Brandao F (1950). Acerca da accao das sulfamidas e das sulfonas na doenca de Duhring. Trab Soc Port Dermatol Venereol.

[17] Clarindo M, Possebon A, Soligo E, Uyeda H, Ruaro R, Empinotti J (2014). Dermatitis herpetiformis: pathophysiology, clinical presentation, diagnosis and treatment. An Bras Dermatol.

[18] Antiga E, Caproni M (2015). The diagnosis and treatment of dermatitis herpetiformis. Clin Cosmet Investig Dermatol.

[19] Egan CA, O’loughlin S, Gormally S, Poweli FC (1997). Dermatitis herpetiformis: a review of fifty-four patients. IJMS.

[20] Caproni M, Bonciolini V, D’Errico A, Antiga E, Fabbri P (2012). Celiac disease and dermatologic manifestations: many skin clue to unfold gluten-sensitive enteropathy. Gastroenterol Res Pract.

[21] Fry L (1982). The treatment of dermatitis herpetiformis. Clin Exp Dermatol.

[22] Piamphongsant T, Ausawamongkonpan S (1976). Bullous pemphigoid controlled by dapsone. Dermatologica.

[23] Piamphongsant T (1983). Dapsone for the treatment of bullous pemphigoid. Asian Pacific J Allergy Immunol.

[24] Bouscamt F, Chosidow O, Picard-Dahan C (1996). Treatment of bullous pemphigoid with dapsone: retrospective study of thirty-six cases. J Am Acad Dermatol.

[25] Venning VA, Millard PR, Wojnarowska F (1989). Dapsone as first line therapy for bullous pemphigoid. Br J Dermatol.

[26] Piamphongsant T (1976). Pemphigus controlled by dapsone. Br J Dermatol.

[27] Haim S, Birnbaum RF (1978). Dapsone in the treatment of pemphigus vulgaris. Dermatologica.

[28] Baum S, Debby A, Gilboa S, Trau H, Barzilai A (2016). Efficacy of dapsone in the treatment of pemphigus vulgaris: a single-center case study. Dermatology.

[29] Tan HH, Tay YK (2000). An unusual case of pemphigus vulgaris presenting as bilateral foot ulcers. Clin Exp Dermatol.

[30] Zuidema J, Hilbers-Modderman ESM, Merkus FWHM (1986). Clinical pharmacokinetics of dapsone. Clin Pharmacokinet.

